# A Preliminary Analysis of Tympanometric Parameters in a Local Multiethnic Population

**DOI:** 10.3390/audiolres10020013

**Published:** 2020-12-16

**Authors:** Kenneth Chua Wei De

**Affiliations:** Department of Allied Health, Audiology, Otorhinolaryngology, Changi General Hospital, Singapore 529889, Singapore; kenneth_chua@cgh.com.sg; Tel.: +65-693-652-59

**Keywords:** acoustic impedance tests, audiology, population groups, gender

## Abstract

*Background*: Tympanometry is a routine clinical test ordered at the Department of Otolaryngology, Ear, Nose and Throat (ENT) at Changi General Hospital (CGH). In combination with the pure tone audiogram, tympanograms aid in the diagnostic value of various middle ear disorders. However, its diagnostic value depends on the physician and audiologist’s accuracy of classifying and interpreting the tympanograms. Presently, Caucasian normative values are used in the classification of tympanograms, which could be inaccurate without population specific norms. Therefore, there is a need to understand ethnic differences in tympanometry parameters in order to usefully interpret the tympanogram. Thus far, there are no local studies on the differences in tympanometric parameters among multiethnic groups. Previous studies also had conflicting results on the effects of ethnicity with direct or indirect comparison only between two ethnic groups. To our knowledge, this is the first preliminary investigation on the effects of demographic and anthropometric measurements on tympanometric parameters. *Materials and Methods*: 90 patients’ medical charts were randomly selected and reviewed to extract demographic, anthropometric and clinical information. Tympanogram characteristics among ethnic groups were investigated using univariate and multivariate analyses. The mean ages of males and females in the study were 41.9 years ± 17.4 and 46.1 ± 19.2, respectively. *Results*: Gender significantly influenced ear canal volume (ECV). Chinese had marginally significant lower static admittance (SA) as compared to non-Chinese. There were, however, no effects of age or anthropometric measurements on tympanometric results. Conclusion: Further prospective large cohort analyses are warranted to expand this investigation to better elucidate differences observed in tympanometric parameters and establish population specific norms for appropriate and accurate tympanogram classifications.

## 1. Introduction

Tympanometry is a quick, noninvasive, and objective procedure to assess the function of the middle ear. It is routinely included in clinical audiology as part of a test battery with pure tone audiometry and is sensitive at identifying various middle ear disorders. In the adult population, the low frequency 226 Hz pure tone is used as the test frequency with varying air pressure in the ear canal [1]. The tympanogram is quantitative, providing data in absolute units and values which include static admittance (SA), tympanic peak pressure (TPP), tympanic width (TW) and ear canal volume (ECV). An audiologist using developed specific norms for the Caucasian population usually interprets the tympanometric parameters [2,3,4]. This is a concern, as past studies have shown us that there are age [5], ethnic [6] and gender [7] effects on tympanometric parameters. If the interpretation of quantitative tympanogram has significant clinical values in the diagnosis of middle ear problems [8], applying the Caucasian norms may not be appropriate, especially for a multiethnic population in Singapore. There are, unfortunately, no population-specific norms for our local multiethnic population yet. To our knowledge, this is the first preliminary analysis of tympanometric parametersin a multiethnic population, which examined the effect of ethnicity and gender on immittance measurements such as SA, TPP, TW and ECV. This study will lay the groundwork for the eventual development of population-specific tympanometry norms.

## 2. Methods

### 2.1. Study Design and Participants

Ninety participants (180 ears—90 right ears, 90 left ears) were randomly selected between January 2017–November 2018. Ethical approval was obtained from the SingHealth Centralised Institute Review Board 2018/2623 (CIRB), Changi General Hospital (CGH), Singapore. This was a retrospective study with demographic, anthropometric and clinical data retrieved from the Sunrise Clinical Manager (SCM) medical records. The study sample included results from adults with hearing within the normal limits or sensorineural hearing loss, when they first presented to the Ear, Nose, and Throat department at CGH and were referred for routine pure tone audiometry and tympanometry. All subjects included were extensively screened by an ear, nose and throat (ENT) physician with unremarkable external or middle ear findings with a negative neurotologic history, and up to a maximum of 10 decibel (dB) acceptable difference in air and bone conduction thresholds on the audiogram for the ears tested. Participants who did not meet these criteria were excluded from the analysis. We extracted information such as age, gender, race, height, weight, body mass index (BMI), body surface area (BSA) and clinical audiometric and tympanometric audiological information.

### 2.2. Procedure

Pure tone air and bone conduction audiometry was conducted at all clinically relevant octave frequencies (250 Hz to 8 KHz) using a Madsen Itera II clinical audiometer. Audiometers were calibrated as specified by ANSI standards [9] with validated permissible noise levels [10] in the audiometric test booths. A handheld Maden Otoflex 100 clinical tympanometer was used in all participants whose data was selected for analysis. Daily calibration of the tympanometer with a 2-cc cavity was performed to ensure that the equipment was performing up to the manufacturer-specified standards prior to performing the test on the participants. The conventional 226 Hz pure tone with pump speed set to medium (200 daPa/s) and pressure range from positive +300 daPa to negative −300 daPa were selected. The recordings were taken once in each ear for every participant, but repeatedly measured if warranted, such as when there was a “B” tympanogram or if the ear canal volume was too low, to ensure validity and accuracy of the test. The measures of SA, TPP, TW and ECV could then be obtained from the tympanometry. SA refers to the peak admittance of the middle ear. TPP refers to the pressure point that corresponds to the maximum height of the tympanogram, which is suggestive of the middle ear pressure at which the system is in optimal compliance and most sound energy is absorbed with correspondingly little sound energy reflected back to the external ear, recorded by the probe. TW is the width of the tympanogram measured at half the peak of the tympanogram, while ECV is the height of the tympanogram measured to give us an estimate of ear canal volume.

### 2.3. Data Analysis

Statistical Package for the Social Sciences (SPSS) version 20 was used for data analysis. Exploratory data analysis was first performed to check for assumptions of normality and equal variances. Levene’s test for equal variances and the Shapiro–Wilk test of normality suggested that SA and TPP had unequal variance (*p* > 0.05) and normality distribution; we analysed the effects of gender and ethnicity on all tympanometry parameters using a nonparametric Wilcoxon signed ranked test for SA and TPP and parametric independent sample t-tests for TW and ECV. Parametric t-tests, analysis of variance (ANOVA) or nonparametric equivalent tests such as the Krustal–Wallis ranked tests were conducted based on the assumptions fulfilled. Univariate and multivariate analyses were conducted to investigate the effects of ethnicity, gender, demographic and anthropometric data on tympanometric parameters.

## 3. Results

There were 54 males and 36 females in total. Of the 90 participants, 66 were Chinese and 24 were non-Chinese. 73.3% (66/90) of the patients were Chinese, 13.3% (12/90) were Malay, 5.6% (5/90) were Indian and 7.8% (7/90) were other races (Phillipino, Sikh, Thai, Burmese and Eurasian). Despite unequal sample size in each ethnic group, this was the demographic ethnic profile of the population at large, with 76.2% Chinese, 15% Malays and 7.4% Indians [11]. Descriptive statistical data for the measured tympanogram characteristics (SA, TPP, TW and ECV) of all the participants are shown in Table 1.

Comparative analysis of age between gender was not significantly different (*p* = 0.28). The mean age of females was 46.1 years ± 19.2 and of males it was 41.9 years ± 17.4. Even among the different ethnic groups, mean age was also not significantly different. The mean age for Chinese was 44.8 ± 17.9; mean age of 39.6 ± 20.3 for Indian; mean age of 36.6 ± 14.2 for Malay and 46.9 ± 25.3 for other races. As the numbers of Malay, Indian and other races were low, we categorized them as non-Chinese for further quantitative comparison with the main Chinese ethnic group.

As the paired t-test did not suggest a significant difference in tympanogram parameters between the ears (*p* > 0.05), the binaural data were averaged between the ears for further analyses. Two-way MANOVA to investigate the effects of gender and ethnicity on tympanogram characteristics was carried out. The dependent variables were SA, TPP, TW and ECV values. Both gender (F (4,83) =3.19, *p* = 0.017; *p* < 0.05) and ethnicity (F (4,83) =3.59, *p* = 0.009; *p* < 0.01) effects were significant in influencing tympanogram results. Further univariate analysis revealed significant differences only for averaged ECV volume between gender (*p* = 0.004; *p* < 0.01) and averaged SA between ethnic groups (*p* = 0.003; *p* < 0.01), with an average of 0.13 cc higher ECV in males and 0.24 mmho higher averaged SA in non-Chinese. When ethnic grouping was stratified by gender and analyzed, it was not surprising that its effect on tympanogram parameters was significant (F(12, 29.4) = 2.652, *p* = 0.015), with influence on SA (*p* = 0.047) and ECV (*p* = 0.006; *p* < 0.01). Post hoc analysis suggested that non-Chinese males (NCM) had on average 0.22 mmho (2 dp) higher SA as compared to Chinese females (CF) with significance (*p* = 0.047). Regardless of gender, non-Chinese (*p* = 0.038; non-Chinese females (NCF), *p* = 0.014; NCM) appeared to have a higher averaged SA as compared to Chinese males (CM) with 0.26 mmho (2 dp) and 0.25 mmho (2 dp) higher averaged SA respectively. Non-Chinese males had on average 0.25 cc higher ECV as compared to Chinese females with a significance of *p* = 0.022.

### 3.1. Ethnicity (Chinese Versus Non-Chinese)

Ethnicity was only significant in influencing SA at a *p* value of 0.007 with an average of 0.24 mmho greater SA seen in non-Chinese (90% range 0.33–1.60) as compared to Chinese (0.25–1.11), and marginally significant for ECV volume (*p* = 0.051), with non-Chinese having a 0.11 cc larger ECV as compared to their counterparts.

### 3.2. Gender (Males Versus Females)

Gender, on the contrary, only had a significant effect on ECV volume (*p* = 0.009), with males on average having a larger ECV by 0.13 cc as compared to Females.

### 3.3. Ethnicity Stratified by Gender (Chinese Females (CF), Chinese Males (CM), Non-Chinese Females (NCF) and Non-Chinese Males (NCM))

When we looked at the distribution of different ethnic groups in non-Chinese (Malay, Indian and others) and compared it with the main Chinese group, the effect was only less significant (*p* = 0.065) for SA but still highly significant for ECV (*p* = 0.006). Further pairwise post hoc analyses revealed that NCM had on average 0.25 cc greater ECV as compared to CF (*p* = 0.004). Analysis of variance (ANOVA) post hoc Tukey HSD analyses showed that Indians had significantly higher ECV volume of 0.38 cc (*p* = 0.002) and 0.42 cc (*p* = 0.008) as compared to Chinese and other races, respectively. This prompted a look into the relationship between anthropometric measures and tympanogram results, as it has been suggested before that different body sizes may affect ECV. Of the 90 participants, only 43 had a complete record of BSA, BMI, height and weight. These anthropometric data were, however, not statistically different in their mean values across the different ethnicity and not statistically significant in multivariate analyses in its influence on tympanogram results.

## 4. Discussion

The present study found significant differences only in ECV and SA, with gender and ethnicity playing a role in influencing ECV and SA, respectively. Gender had a small but significant effect with a larger ECV of 0.13 cc observed in males. Non-Chinese also had greater SA of 0.24 millimhos (mmho is the standard unit of measurement for compliance in immittance testing) and 90% range. When both gender and ethnicity were looked at together, only ECV was highly significant, with non-Chinese males having an average of 0.25 cc larger ECV as compared to Chinese females, but SA differences were only marginally significant. The role of gender in influencing ECV has been elucidated by several studies, and supporting the notion that males generally have a larger body size and hence a larger ear canal [3,6,7,12]. However, past studies have not directly compared anthropometric measures such as height, weight, BSA and BMI between gender or ethnic groups. We considered anthropometric data in this present study; however, it appears that there are other factors unaccounted for that may have influenced ECV, as the anthropometric measures were not statistically significant. This may include different anatomic skull sizes, which was not directly evaluated with measurements of head circumference. BSA and BMI are arguably also not accurate reflections of anatomical body sizes, as there is also an effect of metabolic mass. However, the effects of anthropometric measures have not been investigated before in a local population with multiethnic groups. The effect of gender on ECV is small, but statistically consistent with reports from other studies, which looked at gender differences in the same ethnic group [6,12]. Others had conflicting reports on the effect of ethnicity (between two different ethnic groups) on tympanometric results. This study, however, looked at more than three different ethnic groups (Chinese, Malay, Indians and other races), and when both ethnicity and gender were considered together, there appeared to be a larger effect on ECV [3,6,7,12].

In a study done by Li et al., no significant differences in tympanometric results were observed between the Chinese and Non-Chinese. However, the study only compared the Chinese with Malay adults, who are both of Asian origin and hence have body sizes that may not differ much. When more ethnic groups were included, the effects of ethnicity on SA were, on the contrary, significant. However, this significance became marginal when both gender and ethnicity were looked at together. This could be due to the unequal sample sizes among ethnic groups with only twelve Malays, five Indians and seven other races. Shahnaz and Davis reported that the Chinese had lower SA, wider TW, more positive TPP and lower ECV as compared to the Caucasian population, while Manchaiah and Durisala only agreed on the lower SA and ECV when comparing older Chinese with Caucasians. In this study, there seemed to be an effect of ethnicity on both SA and ECV, which supports the fact that applying tympanometry normative data without specific gender or ethnic considerations may not be appropriate in our multiethnic population. This study also notes no significant effect of age on the different tympanometric parameters, unlike the study done by Manchaiah et al. The differences may be explained by a limited age band (60–90) in the previous study or the effects of age on the middle ear occurring beyond 60 years of age.

Where ECV is concerned, it has less clinical relevance as compared to SA. When an abnormally low ECV is suspected, it could suggest a stenotic/collapsing ear canal, significant outer ear obstruction or blockage of probe tip (instrument blockage, typically probe tip in contact with ear canal wall). These can be easily verified with an otoscopic examination or repositioning of the probe. However, SA out of the normative range may suggest a stiffer property of the middle ear, like in osteosclerotic ears (abnormally low SA) [13] or a discontinuity of the middle ear ossicles (abnormally high SA) post head trauma [14].

## 5. Conclusions

In conclusion, there was a significant relationship between gender and ECV, which is a consistent finding in the literature. Ethnicity also appears to be an important factor, with marginal significance but conflicting results among studies. Applying ethnic specific norms to a group of surgically-confirmed otosclerosis may result in improved overall tympanometry test performance when compared with using norms of other ethnic groups. Further prospective studies with greater representation of participants from different ethnic groups are warranted to better elucidate the effects of ethnicity on tympanometric parameters. There is a need to establish ethnic and gender-specific normative data for tympanometry in larger cohort studies, especially in heterogeneous cosmopolitan Singapore with multiple ethnic groups. Applying non-population-specific normative data may reduce diagnostic confidence and accuracy because of these differences in tympanometric parameters observed. One of the limitations of this study is the small sample size, which can be expanded prospectively in a larger cohort study on establishing population-specific norms on tympanogram parameters. The presence of confounding factors or the absence of some information about some clinical characteristics such as anthropometric data of the sample could represent a limitation. 

## Figures and Tables

**Table 1:** Mean, standard deviation (SD), 95% confidence intervals (CI) and 90% range of tympanometric results by ethnic grouping and gender.

Tympanometric Parameter	Chinese		Non-Chinese		Ethnic Group and Gender
	Male (*n* = 39)	Female (*n* = 27)	Male (*n* = 15)	Female (*n* = 9)	
SA (mmho)					SA between ethnic group *p* < 0.01
Mean ± SD	0.55 ± 0.27	0.58 ± 0.26	0.80 ± 0.40	0.80 ± 0.44	
95% CI	0.45–0.65	0.46–0.70	0.64–0.96	0.60–1.01	
90% Range	0.19–1.16	0.28–1.11	0.33–1.66	0.35–1.42	
TPP (daPA)					*p* > 0.05 between ethnic groups or gender
Mean ± SD	−18.68 ± 21.2	−12.87 ± 14.5	−11.33 ± 11.83	−6.67 ± 9.01	
95% CI	−24.13 to–13.23	−19.42 to–6.32	−20.12 to–2.55	−18.01 to 4.67	
90% Range	−61.0 to 5.0	−57.5 to 2.5	−42.5 to 10	−25.0 to 7.5	
TW (daPA)					*p* > 0.05 between ethnic groups or gender
Mean ± SD	81.61 ± 17.75	76.07 ± 17.36	76.20 ± 15.42	69.11 ± 15.38	
95% CI	76.19–87.05	69.55–82.60	67.45–84.96	57.81–80.41	
90% Range	53.5–114.0	38.5–109.8	53.0–105.5	45.5–90.5	
ECV (cc)					ECV between gender *p* < 0.01
Mean ± SD	1.25 ± 0.23	1.16 ± 0.20	1.41 ± 0.24	1.17 ± 0.20	
95% CI	1.18–1.32	1.07–1.24	1.29–1.52	1.02–132	
90% Range	0.83–1.75	0.86–1.59	1.00–1.72	0.91–1.50	

static admittance (SA), tympanic peak pressure (TPP), tympanic width (TW) and ear canal volume (ECV).
